# Relationship Between Hypertension and Cognitive Function in an Elderly Population: A Population-Based Study in Rural Northern China

**DOI:** 10.3389/fneur.2022.885598

**Published:** 2022-05-16

**Authors:** Jie Bao, Jie Liu, Zhiying Li, Zhen Zhang, Xiao Su, Jiayi Sun, Jun Tu, Jinghua Wang, Jidong Li, Yijun Song, Xianjia Ning

**Affiliations:** ^1^Department of Rehabilitation Medicine, Tianjin Medical University General Hospital, Tianjin, China; ^2^Department of Neurology, Tianjin Medical University General Hospital, Tianjin, China; ^3^Laboratory of Epidemiology, Tianjin Neurological Institute, Tianjin, China; ^4^Key Laboratory of Post-Neuroinjury Neuro-Repair and Regeneration in Central Nervous System, Tianjin Neurological Institute, Ministry of Education and Tianjin City, Tianjin, China; ^5^Center of Clinical Epidemiology and Evidence-Based Medicine, Tianjin Jizhou People's Hospital, Tianjin, China; ^6^Department of Acupuncture, First Teaching Hospital of Tianjin University of Traditional Chinese Medicine & National Clinical Research Center for Chinese Medicine Acupuncture and Moxibustion, Tianjin, China; ^7^Department of Cardiology, Tianjin Medical University General Hospital, Tianjin, China; ^8^Department of Neurosurgery, Tianjin Jizhou People's Hospital, Tianjin, China; ^9^Department of General Medicine, Tianjin Medical University General Hospital, Tianjin, China

**Keywords:** hypertension and cognitive function in elderly population hypertension, cognitive function, elderly, epidemiology, risk factors

## Abstract

The burden of cognitive impairment and dementia is particularly severe in low- and middle-income countries. Although hypertension is an important risk factor for cognitive impairment, the influence of different hypertension classification on cognitive impairment remains controversial. To explore the impact of hypertension and hypertension classification on cognitive function, this study was based on a low-income population aged over 60 years in northern China. This population-based, cross-sectional study was conducted from April 2014 to January 2015 in rural areas of Tianjin, China. A total of 1,171 participants aged ≥ 60 years were included. Participants were interviewed by professional researchers face-to-face, using the pre-designed questionnaire. Cognitive function was assessed using the Mini-mental State Examination (MMSE). Multivariate regression analysis was used to calculate the odds ratio (OR) value. There was a significant association between hypertension and cognitive impairment (OR, 1.415; 95% CI: 1.005–1.992; *P* = 0.047) and a significant positive association between stage 3 hypertension (OR, 1.734; 95% CI: 1.131–2.656; *P* = 0.012) and the prevalence of cognitive impairment. To prevent dementia, clinicians should consider the cognitive function and blood pressure control of low-income individuals aged over 60 years with hypertension in northern China, especially those with stage 3 hypertension. In addition, the inconsistent effects of blood pressure on different cognitive functions should also be considered; special attention should be paid to orientation and concentration.

## Introduction

Hypertension is a major risk factor for cognitive decline and dementia (1). However, the claim that lowering blood pressure reduces the risk of cognitive impairment remains controversial. Some studies report that controlling blood pressure can effectively reduce the risk of cognitive decline (1, 2). Regardless, using a systolic blood pressure target of <120 mmHg in patients with hypertension does not significantly reduce the risk of dementia compared to that of 140 mmHg (3). Similarly, the Secondary Prevention of Small Subcortical Strokes Trial (SPS3 trial) indicated that short-term blood pressure-lowering treatment does not improve the cognitive function of patients with lacunar stroke aged over 60 years (4). Previous studies have also shown a U-shaped relationship between blood pressure and cognition, with either high or low blood pressure adversely affecting cognition (5, 6). Accordingly, the relationship between blood pressure and brain health is complex. The age of hypertension onset and the use of antihypertensive drugs can both affect the impact of blood pressure on cognition (2, 7, 8). The above studies suggest the need for further research regarding the impact of blood pressure classification on cognition.

Moreover, more than 50 million people worldwide suffer from dementia, and the number of patients is expected to triple by 2050 (9). Notably, about two-thirds of individuals with dementia live in low- and middle-income countries, which exerts a heavy medical burden for these countries (10). Furthermore, the prevalence of hypertension is higher in low- and middle-income countries (1), whereas the awareness rate and control rate of hypertension is low (11, 12).

Thus, this study focused on a low-income population in China to study the impact of hypertension classification on cognitive function. These findings will provide some theoretical basis regarding the control of hypertension for the prevention of dementia in low- and middle-income countries.

## Materials and Methods

### Patient Selection

This was a population-based, cross-sectional study conducted from April 2014 to January 2015 in rural areas of Tianjin, China. The participants were from a sub-cohort of the Tianjin Brain Study (13, 14), which has been described previously. Briefly, approximately 95% of the individuals in this study reported a 2014 disposable income per capita of <1,600 USD (15). All residents aged 60 years and older without vision or auditory dysfunction were recruited. Individuals with history of myocardial infarction, stroke, congenital hypophrenia, traumatic brain injury, and mental illness were excluded.

The study was approved by the Ethics Committee at Tianjin Medical University General Hospital to conform to the Declaration of Helsinki regarding use of human subjects (IRB2018-100-01), and written informed consent was obtained from each patient during recruitment.

### Risk Factors and Physical Examinations

The specific information collected and the associated processes have been described in detail (14). Using pre-designed questionnaires, uniformly trained investigators collect participants' information through face-to-face interviews. Demographic information included name, sex, date of birth, and education level; personal history included the presence of hypertension and diabetes and lifestyle factors (including smoking and drinking). Physical examination included height, weight, waist circumference, hip circumference, blood pressure, and heart rate.

Fasting blood glucose, total cholesterol, triglyceride, high-density lipoprotein cholesterol, and low-density lipoprotein cholesterol were measured at the Central Laboratory of Tianjin Jizhou People's Hospital.

### Definitions

Hypertension was defined as a history of hypertension self-reported by the subject and in the medical records of the health center, or the mean systolic blood pressure ≥140 mmHg, diastolic blood pressure ≥ 90 mmHg of two measurements of blood pressure at rest; or the patient was taking antihypertensive drugs (16). Stage of hypertension was defined according to the 2018 Chinese hypertension guideline (17): stage 1, systolic blood pressure 140–159 mmHg or diastolic blood pressure 90–99 mmHg; stage 2, systolic blood pressure 160–179 mmHg or diastolic blood pressure 100–109 mmHg; stage 3, systolic blood pressure ≥180 mmHg or diastolic blood pressure ≥ 110 mmHg or the use of antihypertensive medications. Diabetes was defined as a fasting blood glucose ≥7.0 mmol/L and diagnosed by a superior hospital, taking medication for diabetes, or a self-reported history of diabetes. Smoking was defined as smoking ≥1 cigarette daily for more than 1 year. Alcohol intake was defined as drinking >50 mL of alcohol at least once per week for more than 6 months. Moreover, the participants were categorized into four age groups (60–64, 65–69, 70–74, and ≥75 years) and three educational groups according to the length of formal education [0–5 (illiterate), 6–8 (primary school), and ≥9 years (junior high school and above)].

### Cognitive Function Screening

The Mini-mental State Examination (MMSE) was used to assess the cognitive function of study participants (18). Cognitive impairment was defined in association with education level: MMSE score <17 points in the illiterate group, <22 points in the primary school group, and <26 points in the junior high school and above group (19).

### Statistical Analysis

Continuous variables are described as means and standard deviations. Categorical variables are presented as numbers with frequencies, and the chi-squared test was performed to compare the differences between two groups in the univariate analysis. Risk factors for cognitive dysfunction were studied using multivariate logistic regression analysis, with independent variables selected as statistically significant factors in univariate analyses. A linear regression model was used to test the correlation of multidimensional MMSE score and hypertension (yes or no)/blood pressure classification, and score of multidimensional MMSE as the dependent variable. The independent variables in the regression model were found to be statistically significant in the multivariate analysis as well as to test the correlation of cognitive score and hypertension/blood pressure grading, which was adjusted according to age, sex, education, and drinking history. All statistical analyses were performed using SPSS version 25.0 statistical software (SPSS Inc., Chicago, IL), and a two-sided *P* ≤ 0.05 was considered as statistically significant.

## Results

**Figure 1:**
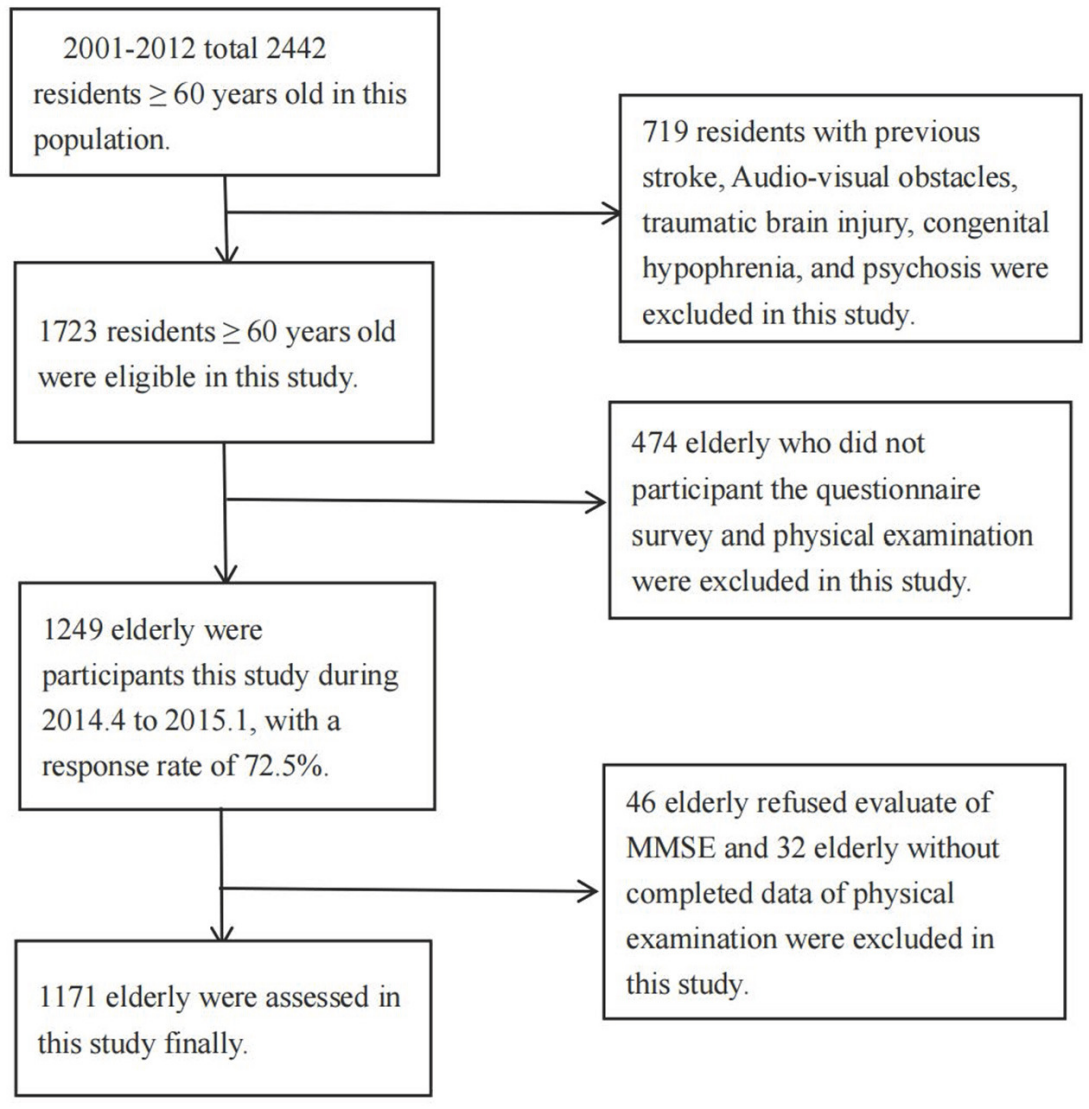
Flow chart of participants. 2001-2012 total 2,442 residents ≥ 60 years old in this population. Eight hundred and thirty nine residents with previous stroke, Audio-visual obstacles, traumatic brain injury, congenital hypophrenia, and psychosis were excluded in this study; 474 elderly who did not participant the questionnaire survey and physical examination were excluded in this study; 46 elderly refused evaluate of MMSE and 32 elderly without completed data of physical examination. Finally, 1,171 elderly were assessed in this study.

During 2001-2012, total 2,442 residents aged ≥ 60 years old were qualified in this population. Of these, 839 residents with previous stroke, Audio-visual obstacles, traumatic brain injury, congenital hypophrenia, and psychosis were excluded in this study; 474 elderly who did not participant the questionnaire survey and physical examination were excluded in this study; 46 elderly refused evaluate of MMSE and 32 elderly without completed data of physical examination. Finally, 1,171 elderly were assessed in this study (Figure 1).

### Baseline Characteristics

Among 1,171 participants, 45.7% were male, and 54.3% were female. The mean age (standard deviation) was 66.84 years overall, 67.31 years for male participants, and 66.64 years for female participants (*P* > 0.05). The average educational level was 3.95 years overall, 5.45 years for male participants, and 2.69 years for female participants (*P* < 0.05). In addition, 60.5% had 0–5 years of education, 29.6% had 6–8 years, and 9.9% had ≥ 9 years; The proportion of residents with no education was 27.8%. The prevalence of cognitive impairment was 32.4% overall, 25.6% for male participants, and 38.1% for female participants. The prevalence of hypertension was 79.4%, diabetes 16.4%, obesity 20.2%, abdominal obesity 34.4%, smoking 58.0%, drinking 29.1%, respectively (Table 1).

**Table 1:** Characteristics of participants in this study.

**Category**	**Men**	**Women**	**Total**	***P*-value**
Total, *n* (%)	535 (45.7)	636 (54.3)	1,171 (100)	
Age, means (SD)	67.3 (6.7)	66.6 (6.8)	67.0 (6.8)	0.09
Age group, *n* (%)				0.10
60~	224 (41.9)	304 (47.8)	528 (45.0)	
65~	138 (25.8)	157 (24.7)	295 (25.2)	
70~	83 (15.5)	72 (11.3)	140 (12.0)	
≥75	90 (16.8)	103 (16.2)	181 (15.5)	
Education, years, mean (SD)	5.5 (2.7)	2.7 (3.0)	4.0 (3.2)	<0.001
Education, *n* (%)				<0.001
0–5 years	215 (40.2)	491 (77.2)	706 (60.3)	
6–8 years	232 (43.4)	109(17.1)	341 (29.1)	
≥9 years	88 (16.4)	36 (5.7)	124 (10.6)	
Smoking history, *n* (%)				<0.001
Never smoke	144 (26.9)	285 (44.8)	429 (36.6)	
Quit smoke	59 (11.0)	4 (0.6)	63 (5.4)	
Smoking at present	332 (62.1)	347 (54.6)	679 (58.0)	
Drinking history, *n* (%)				<0.001
Never drink	277 (51.8)	536 (84.3)	813 (69.4)	
Quit drinking	16 (3.0)	1 (0.2)	17 (1.5)	
Drinking at present	242 (45.2)	99 (15.6)	341 (29.1)	
Hypertension, *n* (%)				0.78
No	112 (20.9)	169 (20.3)	241 (20.6)	
Yes	423 (79.1)	507 (79.7)	930 (79.4)	
Diabetes, *n* (%)				0.01
No	463 (86.5)	516 (81.1)	979 (83.6)	
Yes	72 (13.5)	120 (18.9)	192 (16.4)	
BMI (kg/m^2^), mean (SD)	24.68(3.33)	25.32(3.61)	25.02 (3.50)	0.002
BMI category (kg/m^2^), *n* (%)				0.020
Lightweight	11 (2.1)	14 (2.2)	25 (2.1)	
Normal	218 (40.7)	211 (33.2)	429 (36.6)	
Overweight	216 (40.4)	264 (41.5)	480 (41.0)	
Obese	90 (16.8)	147 (23.1)	237 (20.2)	
Waist (cm), mean (SD)	88.81(9.27)	89.39(8.71)	89.13 (8.97)	0.27
Abdominal obesity, *n* (%)				<0.001
No	491 (91.8)	277 (43.6)	768 (65.6)	
Yes	44 (8.2)	359 (56.4)	403 (34.4)	
SBP, mean (SD)	153.3 (23.0)	154.4 (23.0)	153.9 (23.0)	0.38
DBP, mean (SD)	87.3 (11.5)	85.6 (11.9)	86.4 (11.8)	0.016
FPG, mean (SD)	5.84 (1.16)	6.16 (1.89)	6.01 (1.61)	0.001
TC, mean (SD)	4.57 (0.96)	5.17 (1.06)	4.89 (1.06)	<0.001
TG, mean (SD)	1.43 (0.85)	1.83 (1.29)	1.65 (1.13)	<0.001
HDL, mean (SD)	1.42 (0.44)	1.52 (0.49)	1.47 (0.47)	<0.001
LDL, mean (SD)	2.50 (0.80)	2.84 (0.89)	2.68 (0.87)	<0.001
MMSE scores, median (IQ)	23 (6)	20 (9)	22 (8)	<0.001

*SD, standard deviation; SBP, systolic blood pressure; DBP, diastolic blood pressure; BMI, body mass index; FBG, fasting blood glucose; TC, total cholesterol; TG, triglycerides; HDL-C, high density lipoprotein cholesterol; LDL-C, low density lipoprotein cholesterol; MMSE, Mini-mental State Examination*.

### Influence of Hypertension on Cognitive Impairment in Multivariate Analysis

The multivariate analysis was adjusted for age group, sex, drinking history, and education level, which were associated factors of cognitive impairment in the univariate analysis (Table 2).

**Table 2:** Univariate analysis of risk factors for cognitive impairment.

**Grouping**	**Cognitive function**		***P*-value**
	**Normal**	**Impaired**	
Total:	792	379	
Gender, *n* (%)			<0.001
Men	398 (74.4)	137 (25.6)	
Women	394 (61.9)	242 (38.1)	
Age, means (SD), years	65.7 (5.9)	69.5 (7.8)	<0.001
Education, means (SD), years	4.6 (3.1)	2.6 (3.0)	<0.001
Age group, *n* (%)			<0.001
60~	402 (76.1)	126 (23.9)	
65~	212 (71.9)	83 (28.1)	
70~	97 (62.6)	58 (37.4)	
≥75	81 (42.0)	112 (58.0)	
Smoking status, *n* (%)			0.08
Never smoking	275 (64.1)	154 (35.9)	
Ever smoking	48 (76.2)	15 (23.8)	
Current smoking	469 (69.1)	210 (30.9)	
Alcohol consumption, *n* (%)			0.026
Never drinking	531 (65.3)	282 (34.7)	
Ever drinking	14 (82.4)	3 (17.6)	
Current drinking	247 (72.4)	94 (27.6)	
Hypertension, *n* (%)			0.031
Yes	615 (66.1)	315 (33.9)	
No	177 (73.4)	64 (26.6)	
BP classification, *n* (%)			<0.001
Normal	215 (72.4)	82 (27.6)	
Stage I HBP	293 (72.0)	114 (28.0)	
Stage II HBP	186 (63.9)	105 (36.1)	
Stage III HBP	98 (55.7)	78 (44.3)	
Diabetes, *n* (%)			0.41
Yes	667 (68.1)	312 (31.9)	
No	125 (65.1)	67 (34.9)	
BMI, *n* (%)			0.39
Low weight	18 (72.0)	7 (28.0)	
Normal	277 (64.6)	152 (35.4)	
Overweight	334 (69.6)	146 (30.4)	
Obesity	163 (68.8)	74 (31.2)	
BMI, means (SD), Kg/m^2^	25.11 (3.44)	24.86 (3.61)	0.25
FPG, mean (SD)	5.99 (1.63)	6.06 (1.55)	0.51
TC, mean (SD)	4.86 (1.06)	4.96 (1.06)	0.11
TG, mean (SD)	1.65 (1.18)	1.65 (1.01)	0.93
HDL, mean (SD)	1.46 (0.46)	1.51 (0.5)	0.11

**Figure 2:**
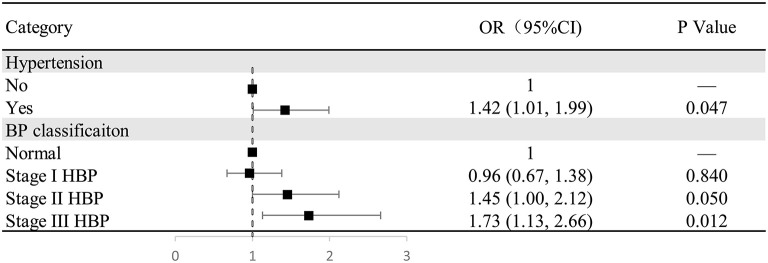
The relationship between hypertension/hypertension classification and cognitive impairment in the multivariate analysis. Figure shows a significant difference between hypertension and cognitive impairment (odds ratio, 1.42; 95% confidence interval: 1.01–1.99; *P* = 0.047). Compared with normal blood pressure, there were no significant differences between stage 1 hypertension (*P* = 0.84) and stage 2 hypertension (*P* = 0.05) and the prevalence of cognitive impairment; in contrast, there was a significant positive correlation between stage 3 hypertension (odds ratio, 1.73; 95% confidence interval: 1.13–2.66; *P* = 0.012) and the prevalence of cognitive impairment.

Figure 2 shows a significant difference between hypertension and cognitive impairment (odds ratio, 1.42; 95% confidence interval: 1.01–1.99; *P* = 0.047). Compared with normal blood pressure, there were no significant differences between stage 1 hypertension (*P* = 0.84) and stage 2 hypertension (*P* = 0.05) and the prevalence of cognitive impairment; in contrast, there was a significant positive correlation between stage 3 hypertension (odds ratio, 1.73; 95% confidence interval: 1.13–2.66; *P* = 0.012) and the prevalence of cognitive impairment.

### Influence of Hypertension on Cognitive Score in Multivariate Analysis

**Figure 3:**
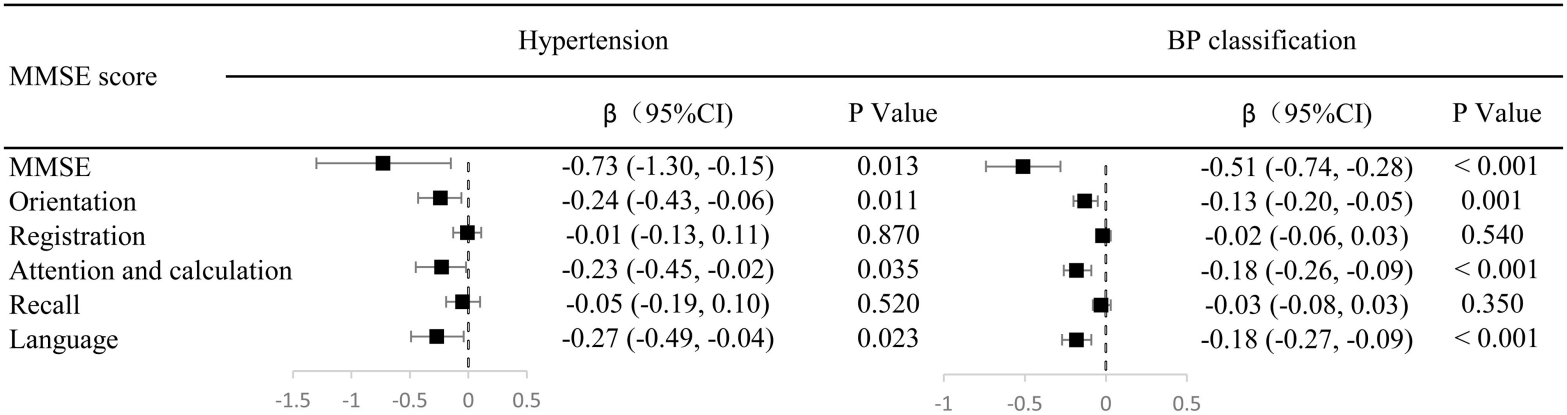
Odd ratios of cognitive function in hypertension and blood pressure grading in multivariate analysis. The hypertension and blood pressure classification were both included in one model, which is mainly adjusted for age group, sex, drinking history, and education. There was a significant association of both hypertension and blood pressure classification with MMSE score (*P* < 0.05). In addition, scores for orientation, attention and calculation, and language significantly decreased by 0.24, 0.23, and 0.27 points, respectively, in the hypertension group compared with the non-hypertension group (all *P* < 0.05). Similarly, compared with the normal blood pressure classification, with each increase in the grade of hypertension, scores for orientation, attention and calculation, and language significantly decreased by 0.13, 0.18, and 0.18 points, respectively, (all *P* < 0.05). However, registration and recall scores were not associated with hypertension and blood pressure classification.

The hypertension and blood pressure classification were both included in one model, which is mainly adjusted for age group, sex, drinking history, and education. There was a significant association of both hypertension and blood pressure classification with MMSE score (*P* < 0.05). In addition, scores for orientation, attention and calculation, and language significantly decreased by 0.24, 0.23, and 0.27 points, respectively, in the hypertension group compared with the non-hypertension group (all *P* < 0.05). Similarly, compared with the normal blood pressure classification, with each increase in the blood pressure classification, scores for orientation, attention and calculation, and language significantly decreased by 0.13, 0.18, and 0.18 points, respectively, (all *P* < 0.05). However, registration and recall scores were not associated with hypertension and blood pressure classification (Figure 3).

## Discussion

This study focused on the relationship of hypertension and blood pressure classification with cognitive function among a low-income elderly population in rural areas of northern China. In this study, the prevalence of cognitive impairment in the hypertension group and the stage 3 hypertension group increased 41.5 and 73.4%, respectively, compared to that in the non-hypertension group. Further analysis showed that compared with the normal blood pressure group, the orientation, attention and calculation, and language scores of the hypertension group decreased by more than 0.2 points. With each increase in hypertension stage, scores for MMSE decreased by more than 0.5 points. The uniqueness of this work is that it is based on a low-income population in northern China and that blood pressure classification were used to further understand the relationship between blood pressure and different cognitive functions.

### Relationship Between Hypertension and Cognitive Impairment

As early as the 1950's and 1960's, some scholars began to systematically study the impact of hypertension on cognitive function (20, 21). Previous studies showed that hypertension can cause a decline in cognitive function, and hypertension with onset in middle ages has the strongest impact on the cognition in elderly individuals (22–25). Consistent with the results of previous studies, compared with individuals with normal blood pressure, the prevalence of cognitive impairment in individuals with hypertension is significantly higher by 41%. The contribution of the present study to the literature is that analyzing by blood pressure classification shows that only stage 3 hypertension is associated with increased prevalence of cognitive impairment. However, the relationship between blood pressure and cognition cannot be generalized, and there may be a non-linear dose-response relationship (26). Another study indicated that among ambulatory adults with hypertension, controlling the target systolic blood pressure below 120 mmHg will not significantly reduce the risk of dementia compared with 140 mmHg (27). In adults with hypertension, decreased renal function as measured by the estimated glomerular filtration rate is associated with increased risk of dementia; nevertheless, intensive hypertension treatment did not reduce the risk (28). This may be related to the relationship between blood pressure and cerebral perfusion, wherein after intensive blood pressure reduction, hypotension and cerebral hypoperfusion may negatively impact the brain cognitive function (29, 30). Therefore, physicians should targeted intervention blood pressure levels in patients with grade 3 hypertension to reduce the negative effects of blood pressure on cognition in an elderly population.

### Hypertension and Different Cognitive Functions

Hypertension is related to almost all areas of cognitive function (31). However, blood pressure affects white matter volume in frontal lobes higher than in occipital, parietal, and temporal lobes (32, 33). Recent studies have shown a larger volume of deep forehead white matter hyperintensities among hypertensive patients (34), which are related to worse scores on cognitive tests of memory (pairs matching), executive function (tower rearranging), and reasoning (matrix pattern) (35). In the current study, the orientation, attention and calculation, and language scores of hypertensive patients were significantly reduced. This may be because different brain tissues have different sensitivity to blood pressure (36), and more in-depth research on the effect of blood pressure grading on brain tissue is necessary.

Hypertension is a major risk factor for vascular dementia (37, 38). Previous studies have shown that changes in blood pressure are associated with changes in cerebral perfusion and metabolism (5, 39, 40). Neurovascular units within the vascular region and the entire brain can respond to changes in blood pressure and increased metabolic demands. But endothelial dysfunction associated with hypertensive patients underlies altered neurovascular coupling and a local decrease in vasomotor reserve (39). Furthermore, increased vascular endothelial oxidative stress and inflammation caused by hypertension lowers the intrinsic threshold for cell survival (39). Therefore, the effect of blood pressure on cognition may be accomplished through multiple pathways.

There are some limitations in this study. Firstly, this study was a single-center cross-sectional study with limited population representation, and the relationship between hypertension and cognition is only a correlation, not a causal relationship. Secondly, this study did not evaluate the effect of hypertension medication on cognitive function, but the rates of awareness of hypertension and of hypertension medications in the study population was low (41), thus the effect of antihypertensive drugs on cognition may be small in this study. Third, in this study, the MMSE score was used to assess the cognitive function of the participants. Although the MMSE did not reveal impaired cognitive function of patients with higher education (42), the study population was low-income and low-educated, with an average education level of just 3.95 years; thus, the screening for cognitive impairment was accurate. Fourth, the effect of hypertension and blood pressure classification on the MMSE score in this study was only 0.5 points. Although there was statistical significance, the clinical significance may not be significant. In clinical work, professional clinicians are required to make judgments based on the actual situation of the patient. Finally, information of quantitative alcohol consumption was lacking of in this study, this may affect the evaluation of the impact alcohol intaking on cognitive. The further quantitative analysis was needed.

## Conclusion

Among a low-income population over 60 years old in northern China, the prevalence of cognitive impairment in hypertensive patients is significantly higher, especially in those with grade 3 hypertension. In addition, in this study, hypertension mainly affects the abilities of orientation, language, and attention and calculation; this may provide some theoretical basis for the prevention of dementia in low- and middle-income countries.

## Data Availability Statement

The raw data supporting the conclusions of this article will be made available by the authors, without undue reservation.

## Ethics Statement

The studies involving human participants were reviewed and approved by the Ethics Committee at Tianjin Medical University General Hospital. The patients/participants provided their written informed consent to participate in this study.

## Author Contributions

XN, YS, and JLi were involved in conception and design, data interpretation for this article, and involved critical review in for this article. JB, JLiu, ZL, ZZ, XS, JS, and JT were involved in data collection, case diagnosis, and confirmation for this article. JB, JLiu, and ZL were involved in manuscript drafting. JW was involved in data analysis for this article. All authors contributed to the article and approved the submitted version.

## Conflict of Interest

The authors declare that the research was conducted in the absence of any commercial or financial relationships that could be construed as a potential conflict of interest.

## Publisher's Note

All claims expressed in this article are solely those of the authors and do not necessarily represent those of their affiliated organizations, or those of the publisher, the editors and the reviewers. Any product that may be evaluated in this article, or claim that may be made by its manufacturer, is not guaranteed or endorsed by the publisher.
